# Transcriptome Sequencing in a Tibetan Barley Landrace with High Resistance to Powdery Mildew

**DOI:** 10.1155/2014/594579

**Published:** 2014-12-22

**Authors:** Xing-Quan Zeng, Xiao-Mei Luo, Yu-Lin Wang, Qi-Jun Xu, Li-Jun Bai, Hong-Jun Yuan, Nyima Tashi

**Affiliations:** ^1^Barley Improvement and Yak Breeding Key Laboratory, Tibet Academy of Agricultural and Animal Husbandry Sciences, Lhasa, Tibet 850002, China; ^2^College of Forestry, Sichuan Agricultural University, Yaan, Sichuan 625014, China

## Abstract

Hulless barley is an important cereal crop worldwide, especially in Tibet of China. However, this crop is usually susceptible to powdery mildew caused by *Blumeria graminis* f. sp. *hordei*. In this study, we aimed to understand the functions and pathways of genes involved in the disease resistance by transcriptome sequencing of a Tibetan barley landrace with high resistance to powdery mildew. A total of 831 significant differentially expressed genes were found in the infected seedlings, covering 19 functions. Either “cell,” “cell part,” and “extracellular region” in the cellular component category or “binding” and “catalytic” in the category of molecular function as well as “metabolic process” and “cellular process” in the biological process category together demonstrated that these functions may be involved in the resistance to powdery mildew of the hulless barley. In addition, 330 KEGG pathways were found using BLASTx with an *E*-value cut-off of <10^−5^. Among them, three pathways, namely, “photosynthesis,” “plant-pathogen interaction,” and “photosynthesis-antenna proteins” had significant matches in the database. Significant expressions of the three pathways were detected at 24 h, 48 h, and 96 h after infection, respectively. These results indicated a complex process of barley response to powdery mildew infection.

## 1. Introduction

Hulless barley (*Hordeum vulgare* L. var.* nudum*) is a diploid (2*n* = 7*x* = 14) monocot and belongs to the family of Poaceae. Hulless barley is also a form of domesticated barley with an easier-to-remove hull. Hulless barley is an important cereal crop worldwide, especially for beer brewing and poultry feed [1]. This crop is often attacked by barley powdery mildew fungus (*Blumeria graminis* f. sp.* hordei*), which is one of the most destructive pathogens of barley. Powdery mildew causes considerable damage and severe loss of grain yield [2]. It is crucial to collect genetic resources resistant to this disease and further identify underlying genes of resistance to powdery resources. In barley, a great number of landraces have been cultivated across the world and present large genetic variation in many desirable traits, including disease resistance. Indeed, most of the genes for resistance to powdery mildew in currently used cultivars were found in barley landraces [3–7]. A few resistance genes against powdery mildew have been studied in barley, such as* mla* [8],* mlo* [9],* mlg* [10],* mlhb* [11], and* mlf* [12]. Nevertheless, many resistance genes have lost their effectiveness as new races of the pathogen have evolved. Hence, discovering new candidate genes in barley genome sequence is of particular importance. To date, the resistance mechanisms for powdery mildew at physiological and gene levels remain unknown, so comprehensive transcriptomic sequencing of barley varieties with the disease resistance may improve our understanding of plant reaction to pathogen infection.

In the past few years, it has been widely demonstrated that high-throughput next generation sequencing technology makes it possible to carry out genome-wide studies of transcriptomes in a cost-efficient way to explore genes and expression profiling of model and nonmodel organisms [13–15]. Transcriptome sequencing and characterization using Illumina II sequencing technology have been successfully used to interrogate transcriptomes of many organisms such as yeast [16, 17], sweet potato [18], rice [19], taxus [20], migratory locust [21], and giant panda [22]. Despite its obvious potential, Illumina second generation sequencing has not been applied to barley variety for powdery mildew resistance analysis.

The present study was undertaken to provide a broad survey of genes associated with barley resistance to powdery mildew, by transcriptome analysis using Illumina technology. The main goals of this work were as follows: (1) to discover new genes related to powdery mildew resistance; (2) to characterize the gene expression profiles during pathogen infection processes; and (3) to reveal the functions and pathways of the genes involved in the disease resistance mechanism.

## 2. Materials and Methods

### 2.1. Plant Materials, Pathogen Infection, and RNA Extraction

Hulless barley cultivar “Gan Nong Da 7,” displaying high resistance to powdery mildew (unpublished work), was used in this study. Barley seeds were sown in the greenhouse. Three weeks later, the seedlings were infected by powdery mildew (isolated from the field infected barley) and then kept in dark at 18°C for 24 hours and finally kept in light for 14 hours every day. Barley leaves were harvested at six growth stages after infection: 0 h, 24 h, 48 h, 72 h, 96 h, and 120 h, respectively (see Supplementary Figure 1 in Supplementary Material available online at http://dx.doi.org/10.1155/2014/594579). More information about the samples can be found out in Supplementary Table 1. For Illumina sequencing, the total RNA of each of the samples was isolated using an RNAiso Plus (TaKaRa, Japan) protocol and then further purified with RNase-free DNase I (TaKaRa, Japan).

### 2.2. cDNA Library Construction and Sequencing

Briefly, Sera-Mag Magnetic Oligo (dT) Beads (Illumina, San Diego, CA) were used to isolate poly (A) mRNA after the total RNA was collected from the leaves. Fragmentation buffer was added for interrupting mRNA into short fragments. Then, by using these short fragments as templates, random hexamer (N6) primers (Illumina, San Diego, CA) were used to synthesize the first-strand cDNA. The second-strand cDNA was synthesized in the buffer containing dNTPs, RNase H, and DNA polymerase I. Paired end library was constructed by the Genomic Sample Prep kit (Illumina, San Diego, CA) according to manufacturer's instructions. Short fragments were purified with QiaQuick PCR extraction kit (Qiagen, Valencia, CA) and resolved with EB buffer for end repair and adding poly (A). After that, the short fragments were connected with the sequencing adapters. For amplification with PCR, we selected suitable fragments (200 ± 25 bp) as templates based on the result of agarose gel electrophoresis. At last, the library was sequenced using Illumina Genome Analyzer IIX (Illumina, San Diego, CA).

### 2.3. Mapping Reads to Reference Genome

The reference genome was downloaded from the Barley Genome Database (http://150.46.168.145/gbrowse_new/). Sequencing-received raw image data was transformed by Base Calling into raw data or raw reads. Raw sequences were transformed into clean tags by removing reads with adaptor contamination, reads of low quality (reads containing *N*s > 10), and the reads with more than 50% *Q* ≤ 5 bases. Then, the saturation analysis was performed to check whether the number of detected genes increased along with sequencing amount (total tag number). The distribution of clean tag expressions was used to evaluate the normality of the whole data. After that, the remaining reads were aligned to the reference genome using software program TopHat 2.0.9 (Johns Hopkins University; see http://ccb.jhu.edu/software/tophat/index.shtml), following the procedure: tophat-p4-library-type, fr-unstranded-G gff.

### 2.4. Normalized Gene Expression Level by RNA-Seq

The expression levels of genes based on RNA-Seq was normalized by the number of reads per kilo base of exon region in a gene per million mapped reads (RPKM) [23]:

(1) $$\begin{array}{c} \text{RPKM}=\frac{10^{6}*R}{\left(N*L\right)/10^{3}}, \end{array}$$

where RPKM is the reads per kilo base transcript per million reads, *R* is the number of mappable reads to a gene, *N* is the total mapped reads in the experiment, and *L* is the sum of the exons in base pairs.

RPKM is able to avoid the difference from gene length and total sequence data effect on gene expression. The cut-off value for determining the background expression level was at 95% confidence interval for all RPKM values of each gene. The results from this formula were directly used to compare the difference in the gene expressions among the samples at different time sequences.

### 2.5. Evaluation of DGE (Differentially Expressed Genes) Libraries

For screening of DGEs between different samples, a rigorous algorithm was developed based on the previous method [24]. *P* value corresponds to differential gene expression test. The threshold of *P* value in multiple tests was determined through manipulating the false discovery rate (FDR) value. We use FDR ≤ 0.05 as the threshold to judge the significance of DGEs.

Gene ontology (GO) terms were analyzed by the software Blast2GO v 2.3.4 [25] using the default parameters. This program was used to obtain the number of each gene term (GO annotation), and then hypergeometric tests were applied to detect GO enrichment analysis of functional significance in DEGs. The calculating formula is

(2) $$\begin{array}{c} P=1-\overset{m-1}{\underset{i=0}{\sum }}\frac{\left(\begin{array}{c} m-1\\ i \end{array}\right)\left(\begin{array}{c} N-M\\ n-i \end{array}\right)}{\left(\begin{array}{c} N\\ n \end{array}\right)}, \end{array}$$

where *N* is number of genes with annotation, *n* is the number of differently expressed genes in *N*, *M* is the number of genes that are annotated to the certain GO term, and *m* is the number of DEGs in *M*.

The Kyoto Encyclopedia of Genes and Genomes (KEGG), the major public pathway-related database, was used in the pathway enrichment analysis to identify significantly enriched metabolic pathways or signal transduction pathways in DEGs compared with the whole transcriptome background. The calculating formula is the same as that in the GO analysis: *N* is the number of all genes with KEGG annotation, *n* is the number of DEGs in *N*, *M* is the number of all genes annotated to the specific pathways, and *m* is the number of DEGs in *M*. The *Q* value of a test measures the proportion of false positives incurred (i.e., false discovery rate) when that particular test is called significant (http://genomics.princeton.edu/storeylab/qvalue/). Pathways with *Q* value ≤0.05 are significantly enriched in DEGs.

## 3. Results

### 3.1. Summary of RNA-Sequencing Data Sets

To obtain a dynamic view of the gene expression profiles of barley powdery mildew resistance at different infection progress stages, six cDNA samples were prepared from barley leaves at 0 h, 24 h, 48 h, 72 h, 96 h, and 120 h after infection. And then these samples were subjected to the Illumina sequencing platform. In total, we acquired more than 42.90 G raw reads over six time points (Table 1). After cleaning the reads with the proportion of *N* over 10%, over half of proportion of base quality *Q* less than 5 bases, and the adapter polluted reads, approximately 39.80 G clean reads were collected, with 96.25% of the *Q* 30 bases (base quality over 30). The average data of each sample was approximately 6.64 G in size. The following data analysis procedures were based on the modified reads.

### 3.2. Evaluation of the Sequencing Data Quality

To assess the quality and coverage of the sequencing data, mean quality distribution and base distribution were analyzed. Sequencing error rate is not only related to base quality but also influenced by sequencer, reagent, sample, and so forth. Each base sequencing error can be judged by *Q*_phred_ (Phred score), which is given by a model of prediction base judging error probability during Base Calling. The sequencing base mean quality distributions of six samples were similar to each other. For example, the mean quality distribution of Sample A (the sample at 0 h after infection, namely, TR130348) was illustrated in Supplementary Figure 2. The base position in reads is aligned as the *x*-axis and the mean *Q*_phred_ as the *y*-axis. High proportion of *Q* 30 reads indicted high-quality sequence.

Base distributions of all samples were also similar and that in Sample A, as an example, was illustrated in Supplementary Figure 3. The base position in reads is aligned as the *x*-axis and the percentage of ATGC base as the *y*-axis. In general, the equal proportions of bases between T and A and between G and C were found, indicating no preference during sequencing. The GC percentage of each sample accounted for approximately 54% of the total.

### 3.3. Mapping Reads Coverage

The mapping results were listed in Table 1. Each of the samples had the mapped read rates greater than 86%, which indicates that most sequencing data are consistent with the reference genome of barley.

**Figure 1:**
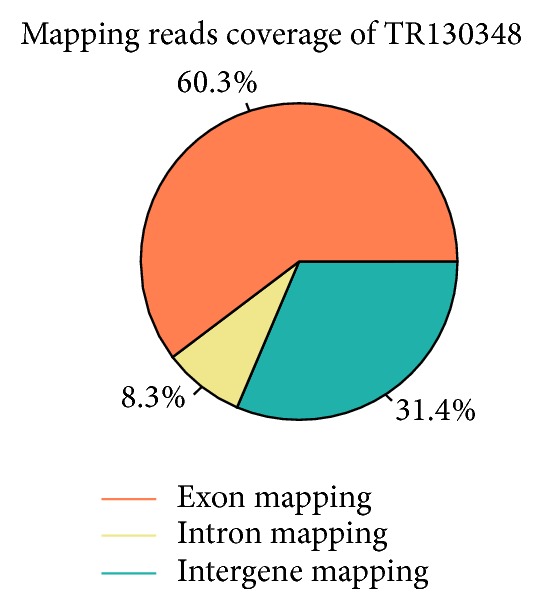
Mapping reads (exon, intron, and intergene) coverage of A (C0, TR130348).

Based on the mapped reads, the proportion of exon mapping, intron mapping, and intergene mapping of sample A at 0 h since infection 5 were illustrated in Figure 1. The highest exon mapping (60.3%) was found in Sample A, while the lowest (52.1%) was found in Sample F (C120, TR130353). The intron mapping coverage ranged from 8.3% (A) to 10.8% (B, C24, TR130349). The average of intergene mapping was 35.2%. There was no affinity with reference genome annotation.

In order to detect the depth of bases, exon gene was divided into 100 parts. The relative positions of genes are aligned as *x*-axis, and the number of reads is aligned as *y*-axis. The line charts of all samples were similar to each other, and Sample A was illustrated in Supplementary Figure 4. There was a little preference of gene exon to the base depth.

### 3.4. Gene Expressions

**Figure 2:**
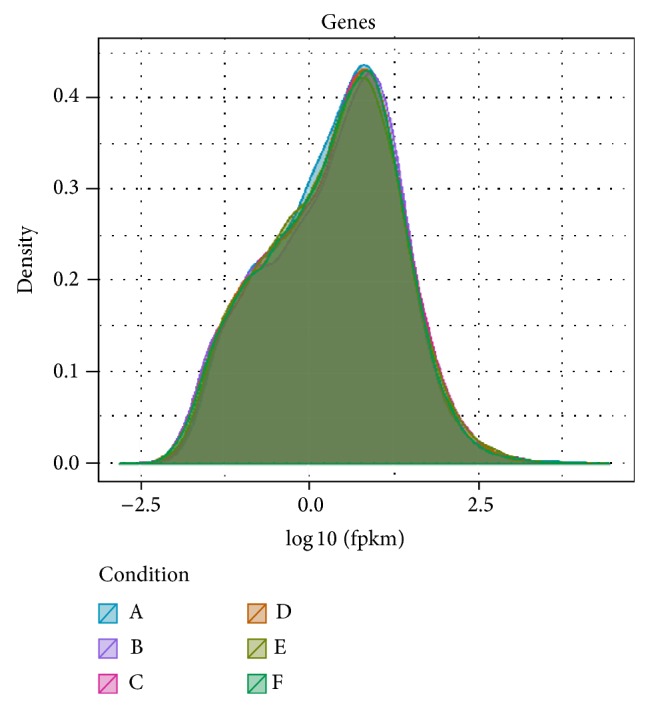
Distributed density of gene global expression of each sample.

**Figure 3:**
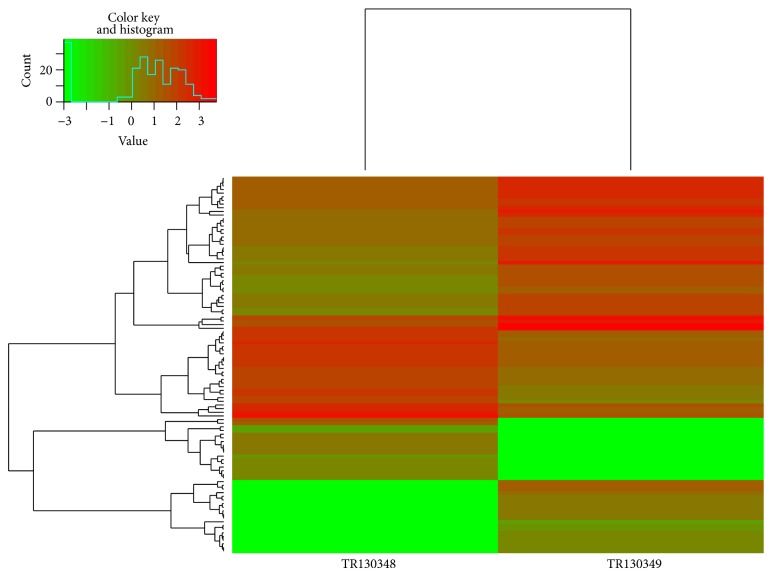
Euclidean distance was used to establish the distance of expression between A (C0, TR130348) and B (C24, TR130349).

Gene saturation of each sample was also similar, and Sample A was illustrated in Supplementary Figure 5. Comparisons of gene expressions in each subsample to the whole sample showed less than 15% of the difference between them, indicating fine expression genes in the current size of sequencing data. The results represented an accurate dataset to detect highly expressed genes. The distribution density of gene global expressions of each sample was illustrated in Figure 2, which exhibits similar expressed gene distributions among these samples. Cuffdiff software (http://cufflinks.cbcb.umd.edu/index.html) was used to compare the gene expressions between sample pairs. Differently expressed genes were identified based on genes with *q* < 0.05 (*q* is the corrected *P* value). The results were listed in Supplementary Table 2. Total DEGs from each sample were clustered in Supplementary Figure 6. The first subgroup contained only A, the second one included B and C (C48, TR130350), and the last one covered the rest samples of D (C72, TR130351), E (C96, TR130352), and F. Euclidean distance was used to estimate the distance of gene expression between sample pairs. The clusters of A and B were illustrated in Figure 3. In the histogram, the red color indicates upregulation and the green color downregulation. Compared with Sample A, gene expressions of B and C were significantly different. There were similar distributions between A and D, between A and E, and between A and F.

### 3.5. Functional Classification by GO

**Figure 4:**
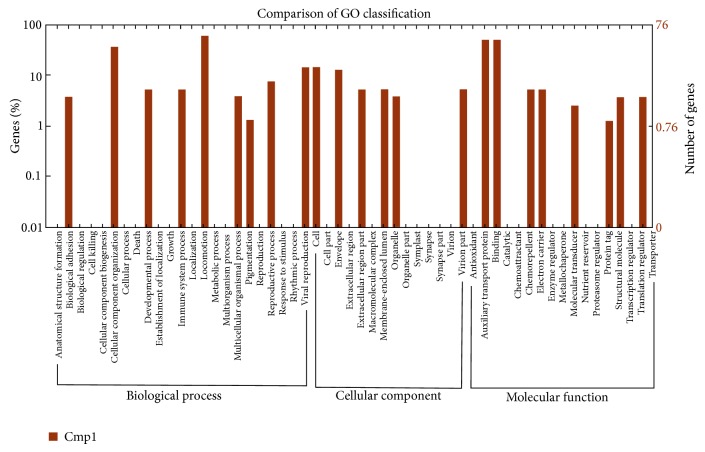
Histogram presentation of gene ontology classification between A (C0, TR130348) and B (C24, TR130349). The results are summarized in three main categories: biological process, cellular component, and molecular function. The right *y*-axis indicates the number of genes in a category. The left *y*-axis indicates the percentage of a specific category of genes in that main category.

GO is an international standardized gene functional classification system which offers a dynamic-updated controlled vocabulary and a strictly defined concept to comprehensively describe the properties of genes and their products in any organism. GO has three ontologies: molecular function, cellular component, and biological process. In total, 39,197 reads with BLASTx matches to known proteins were assigned to gene ontology classes, with 2,654 functional terms. Of them, assignments to the biological process made up the majority (1344, 50.64%), followed by molecular function (1060, 39.94%) and cellular component (250, 9.42%). These functional classifications by GO were summarized in Table 2. Comparison of GO classification between A and B was presented in Figure 4.

A total of 831 significant DEGs were found by Cufflink. The assigned functions of these genes covered a broad range of GO categories. Under the cellular component category, cell, cell part, and extracellular region, including “thylakoid part,” “photosystem,” and “photosystem II,” were prominently represented, indicating that some powdery mildew-related metabolic activities of photosynthesis occurred in the leaf of the Tibetan barley landrace. Interestingly, many genes were assigned to “oxygen evolving complex.” It was also noteworthy that a large number of genes were involved in “extrinsic to membrane.” Under the category of molecular function, binding and catalysis, including “ADP binding” and “auxiliary transport protein,” represented the majorities of the category. Among the genes assigned to auxiliary transport protein, “endonuclease activity” represented the most abundant classification, followed by “ribonuclease activity,” “endoribonuclease activity,” and “ribonuclease T2 activity.” For the biological process category, many genes were classified into the metabolic process and cellular process, including “photosynthesis, light harvesting,” whereas only a few genes were assigned to “defense response,” “response to stress,” and “generation of precursor metabolites and energy.”

### 3.6. Functional Classification by KEGG

KEGG is a public database recording the networks of molecular interactions in the cells and variants of them specific to particular organisms. Pathway-based analysis helps to further understand the biological functions and interactions of genes. First, based on comparison with the KEGG database using BLASTx with an *E*-value cut-off of <10^−5^, 330 KEGG pathways were detected. Among them, three pathways, that is, “photosynthesis,” “plant-pathogen interaction,” and “photosynthesis-antenna proteins,” had significant matches in the database. As shown in Table 2, the “photosynthesis” pathway became distinct at the stage of 24 h after infection, the “plant-pathogen interaction” pathway also differed significantly at the time, and the “photosynthesis-antenna proteins” and “photosynthesis” pathway was remarkable 96 h after infection. These results indicated a dynamic and complex process of barley response to powdery mildew.

## 4. Discussion

### 4.1. Illumina Paired End Sequencing and Assembly

In this study, the mRNA of the barley plants infected with powdery mildew pathogen was sequenced using Illumina Genome Analyzer, with Sera-Mag Magnetic Oligo (dT) Beads. A clear bioinformatic map of mRNA involved in multiple biological processes was produced. As a result, 42.9 G data was collected from six samples over infection time. After filtering, the average data size of each sample was 6.64 G, and the reads number was 66.38 M, which met the requirements for further analysis. Saturability analysis indicated a qualified coverage of most genes based on our data size. In addition, the clean reads of *Q* 30 occupied over 95% of the total, suggesting high-quality sequencing.

TopHat package was used to blast the transcriptome data to the reference genome. It has been found that 86% of the reads were mapped to the reference genome. Multiblasted reads were greater than 10%, which might suggest that they were repeatable in this species. Further analysis of the mapping reads showed that the average of intergene mapping reads was more than 30%, which might be due to inadequate annotation of the genome, as reported by Luo et al. [26].

### 4.2. Functional Annotation of DEGs

On the basis of extensive examination of the DEGs between samples, 831 significant DEGs were found across nineteen functions. These functions were related to cell, cell part, and extracellular region in the cellular component category, binding and catalytic in the category of molecular function, and metabolic process and cellular process in the biological process category. This indicated that these functions were likely involved in powdery mildew-resistant hulless barley. Hulbert et al. [27] summarised that the powdery mildew resistance genes carry motifs found in other receptor and signal transduction proteins, such as nucleotide-binding site domains and kinase domains. Active oxygen in some species has been found to play a number of critical roles in defence responses during plant-pathogen interactions [28–30]. Warren et al. [31] reported that it functioned in defense response signaling of an* Arabidopsis *mutation since it interfered with resistance conferred by several other nucleotide-binding site genes. The cellular components and processes reflect where resistance genes interact with their corresponding elicitors. The cell membrane and extracellular leucine-rich repeats indicated the association between transmembrane domain and the corresponding kinase [32–34]. The observed interaction with intracellular resistance genes products should stimulate researches into how these diverse organisms deliver elicitors into plant cells.

Furthermore, KEGG was used to annotate the DEGs by enrichment analysis and revealed the significant pathways involved in the disease resistance. Three pathways occurred in different stages: the infection firstly acted on “photosynthesis” of leaves and then caused “pathogen recognition interaction” and defense response signaling and finally affected “photosynthesis-antenna proteins.” This event sequence exhibited a dynamic process of barley responding to powdery mildew. Once the recognition of pathogen occurred, the defense responses were triggered. These are often characterized as a hypersensitive response, which involves the death of the first cell or cells infected and the local accumulation of antimicrobial compounds [35].

KEGG analysis revealed that resistance gene action was coupled to a complex series of biochemical defense pathways. It is therefore more likely that resistance genes may function together in recognizing pathogen elicitors, possibly as coreceptors. The similarity in structure of the tomato Cf proteins [36] to the rice Xa21 protein [37] implies that the transmembrane domain genes may also include a kinase in their defense-signaling pathway. Mla resistance protein, containing recognition complexes, may be activated by RAR1/SGT1 (two conserved-interacting proteins in mutants of barley Rar1) [38].* Mlo* resistance genes were triggered by a rapid formation of enlarged cell wall appositions below the fungus's encounter sites and of a physical and chemical barrier that the infection peg can rarely penetrate [9].* Mlo* allele encodes a putative membrane protein, which may be a negative regulator of certain defense responses [39], whereas* Ror* (required for* mlo*-specified resistance) genes act as positive regulators of a non-race-specific resistance response [40]. Barley lines that are homozygous for the nonfunctional alleles show spontaneous defense responses like cell wall appositions in the epidermal cells and even some cell death [41]. Piffanelli et al. [42] inferred that the CIS- (cytokine-induced SH2-containing protein-) dependent perturbation of transcription machinery assembly by transcriptional interference in Mlo-11 plants is a likely mechanism leading to disease resistance.

## 5. Conclusions

This work presents a first report of the transcriptome sequencing of the Tibetan barley landrace with powdery mildew resistance and brings a major genomic resource for barley resistance to this disease. A large number of genes in the hulless barley were characterized by DEG analysis using Illumina sequencing technology. The transcriptome and DEG analyses also provided us with a genome-wide view of the transcriptional mechanisms to improve genome annotation and enabled us to understand some related biological progress of hulless barley disease resistance. The data in this study is consistent with those using multiple approaches including QTL mapping and FISH, indicating the reliability of the results from the mRNA-Seq and DEG analysis. Therefore, further work is needed to find additional linked DNA markers for these DEGs. It is necessary to develop new, reliable, PCR-based markers tightly linked to the resistance genes and this will greatly facilitate gene transfer into currently used varieties.

## Supplementary Material

Two supplementary tables and six figures are available online at http://dx.doi.org/10.1155/2014/594579. Sample information is illustrated in table 1 and figure 1. The evaluation of sequence data is illustrated in figures 2-5. Different gene expression (DGE) is illustrated in table 2 and figure 6.

## Figures and Tables

**Table 1:** RNA-sequencing data filtering analysis.

Library	A^*^	B	C	D	E	F	Average	Total
Original reads number (G)	7.17	6.40	6.88	7.79	7.14	7.51	7.15	42.90
Modified reads number (G)	6.66	5.92	6.38	7.24	6.61	7.00	6.64	39.83
Modified Q30 bases rate (%)	96.22	96.21	96.27	96.19	96.2	96.41	96.25	—
Mapped rate (%)	86.34	86.21	86.99	86.82	86.89	86.55	86.63	—
Multimap rate (%)	12.82	9.82	14.03	12.59	12.81	14.13	12.7	—

^*^A–F: the samples collected at 0 h, 24 h, 48 h, 72 h, 96 h, and 120 h after infection.

**Table 2:** Functional classification by GO and KEGG.

Category	*Q*-value	Function
A_B cellular component		
GO:0044464	0.03867023	Cell part
GO:0005576	3.05*E* − 06	Extracellular region
GO:0044436	0.02673204	Thylakoid part
GO:0009521	0.02559572	Photosystem
GO:0009523	0.01277032	Photosystem II
GO:0009654	0.00933201	Oxygen evolving complex
GO:0019898	0.00908508	Extrinsic to membrane
B_E cellular component		
GO:0005576	0.04652511	Extracellular region
A_C molecular function		
GO:0004519	0.01846397	Endonuclease activity
GO:0004540	0.00563478	Ribonuclease activity
GO:0004521	0.00325892	Endoribonuclease activity
GO:0016894	2.10*E* − 05	Endonuclease activity, active with either ribo- or deoxyribonucleic acids and producing 3′-phosphomonoesters
GO:0016892	2.10*E* − 05	Endoribonuclease activity, producing 3′-phosphomonoesters
GO:0033897	2.10*E* − 05	Ribonuclease T_2_ activity
A_E molecular function		
GO:0016894	0.04079433	Endonuclease activity, active with either ribo- or deoxyribonucleic acids and producing 3′-phosphomonoesters
GO:0016892	0.04079433	Endoribonuclease activity, producing 3′-phosphomonoesters
GO:0033897	0.04079433	Ribonuclease T_2_ activity
C_E molecular function		
GO:0043531	0.01381725	ADP binding
B_E biological process		
GO:0006091	0.01070069	Generation of precursor metabolites and energy
GO:0009765	4.88*E* − 05	Photosynthesis, light harvesting
B_F biological process		
GO:0009765	0.00971227	Photosynthesis, light harvesting
C_E biological process		
GO:0006952	0.00970112	Defense response
GO:0006950	0.00970112	Response to stress
A_B kegg		
map00195	0.0422721	Photosynthesis
A_C kegg		
map04626	0.00085699	Plant-pathogen interaction
B_E kegg		
map00196	6.56*E* − 05	Photosynthesis-antenna proteins
B_F kegg		
map00196	0.00126333	Photosynthesis-antenna proteins
C_E kegg		
map04626	0.00751478	Plant-pathogen interaction
map00196	0.04943251	Photosynthesis-antenna proteins
