# Supplementary material for: Transcriptome Sequencing in a Tibetan Barley Landrace with High Resistance to Powdery Mildew
Source: ScientificWorldJournal. 2014 Dec 22;2014:594579. doi: 10.1155/2014/594579 (PMC4284937; doi:10.1155/2014/594579)
Supplement: Supplementary file 1 — Two supplementary tables and six figures are available online at http://dx.doi.org/10.1155/2014/594579. Sample information is illustrated in table 1 and figure 1. The evaluation of sequence data is illustrated in figures 2-5. Different gene expression (DGE) is illustrated in table 2 and figure 6. [file 594579.f1.pdf]

Supplementray Table 1 Sample information.

Supplementray Table 2 Different gene expression (DGE) of each two samples.

Supplementray Figure 1 Barley leaves at six growth stages after infection (0 h, 24 h, 48 h, 72 h, 96 h, and 120 h).

Supplementray Figure 2 Mean Quality Distribution of A (C0, TR130348). The base position in reads is as the X-axis, and the mean Qphred is as the Y-axis.

Supplementray Figure 3 Base Distribution of A (C0, TR130348). The base position in reads is as the X-axis, and the percentage of ATGC base is as the Y-axis.

Supplementray Figure 4 Gene depth distribution of A (C0, TR130348). The relative position in gene is as X-axis, the number of reads is Y-axis.

Supplementray Figure 5 Gene Saturation of A (C0, TR130348).

Supplementray Figure 6 Total DGEs from each sample was clustered.

Supplementary Table 1 **Sample information.**

Accession no.	Sampling hour	Sample code	Group name
TR130348	0	C0	A
TR130349	24	C24	B
TR130350	48	C48	C
TR130351	72	C72	D
TR130352	96	C96	E
TR130353	120	C120	F

Supplementary Table 2 **Summary of differently expressed genes (DEG).**

Accession	A	B	C	D	E
B	103				
C	93	39			
D	44	42	49		
E	49	102	108	27	16
F	73	29	36	21	

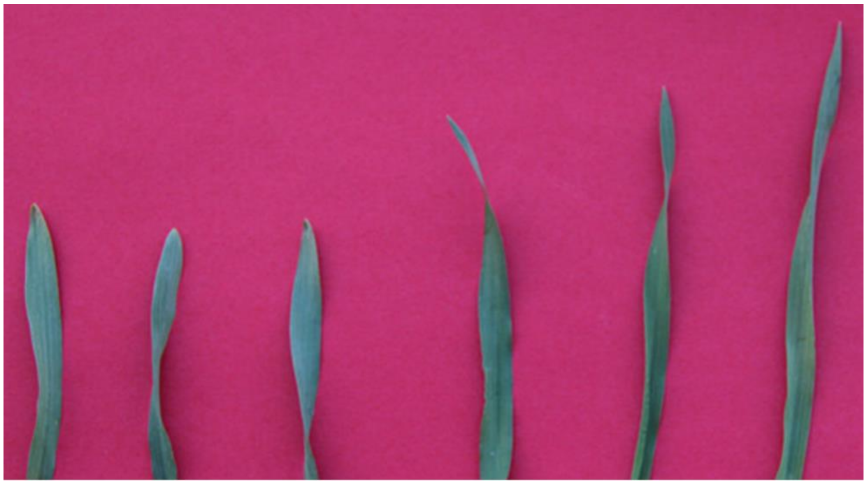

Supplementary Figure 1

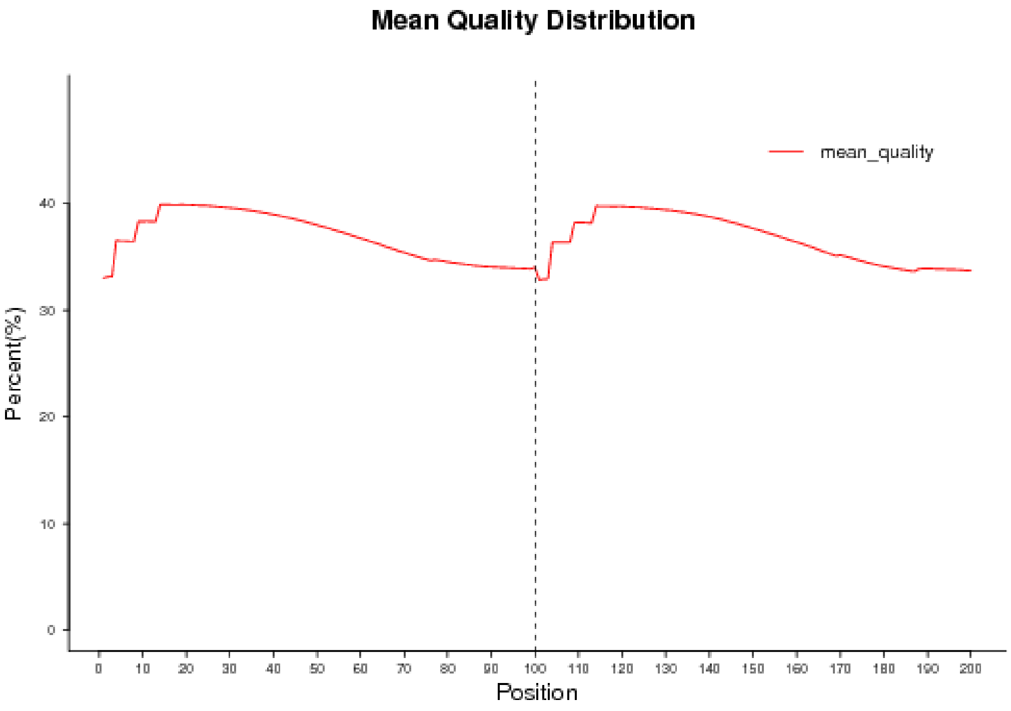

Supplementary Figure 2

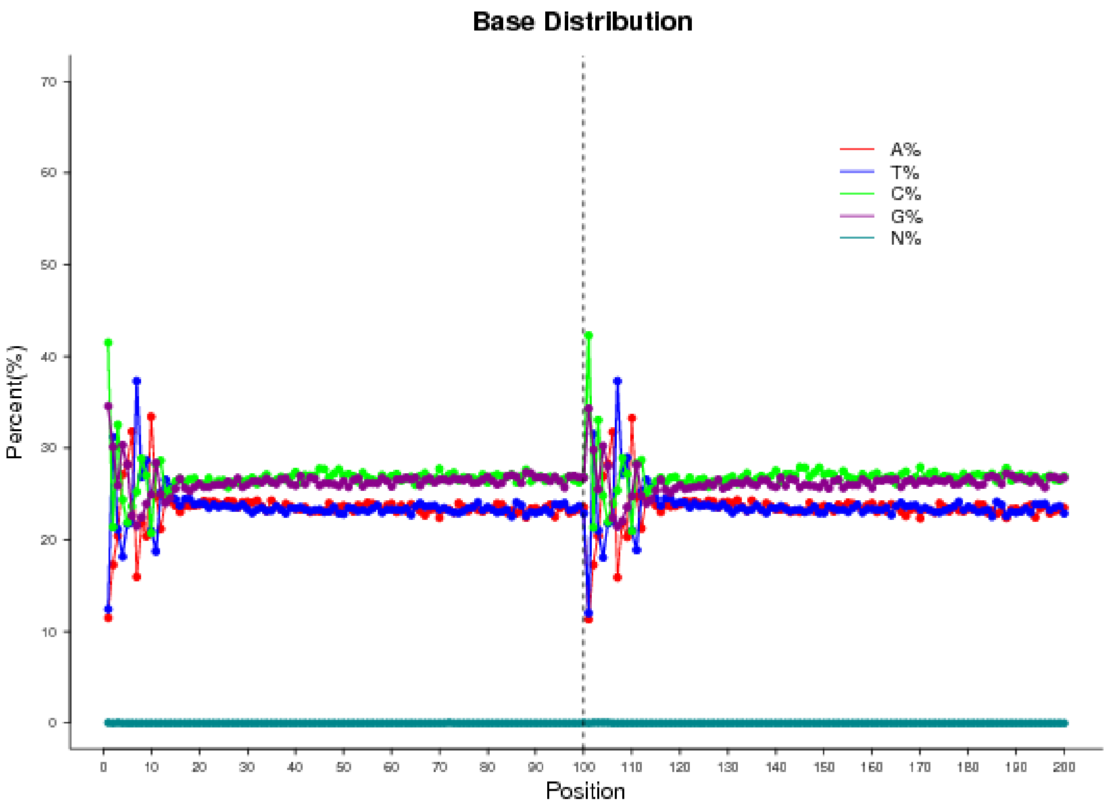

Supplementary Figure 3

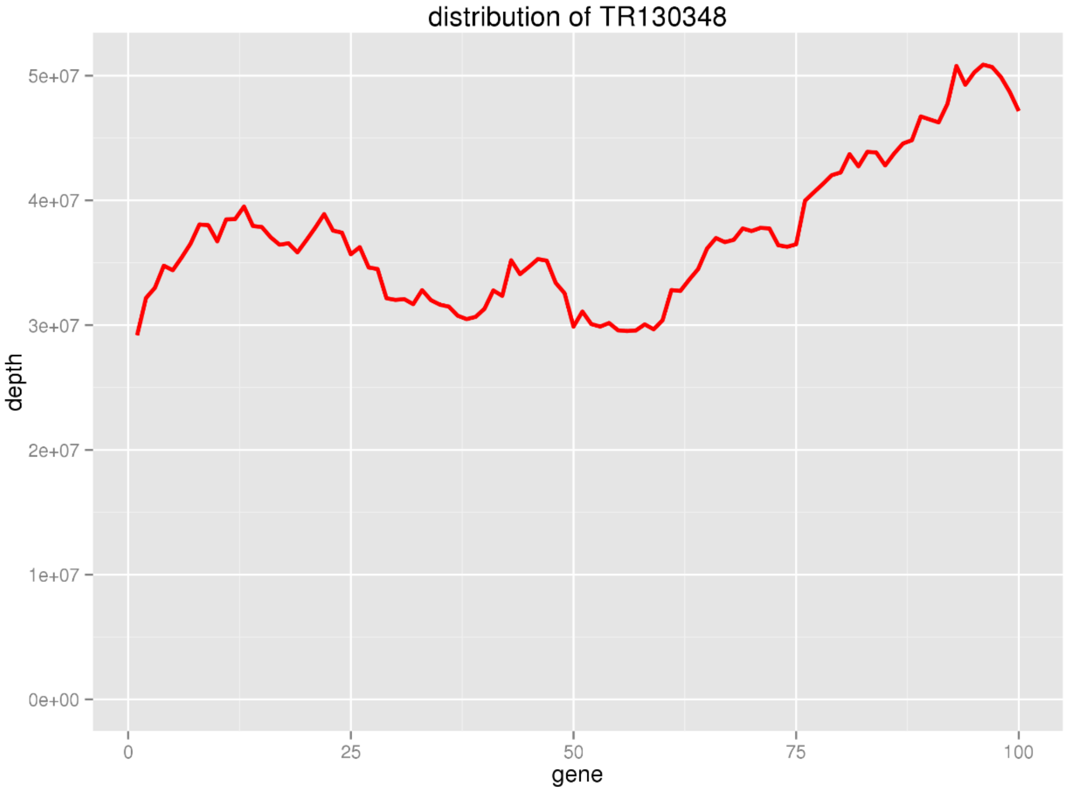

Supplementary Figure 4

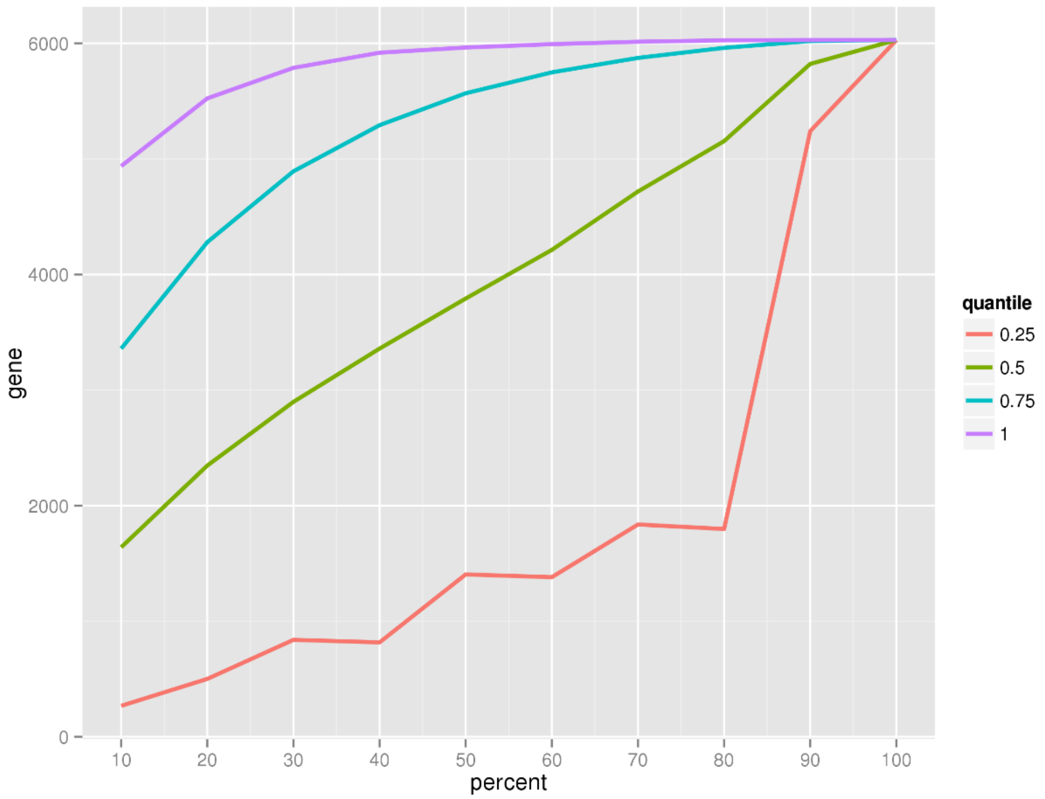

Supplementary Figure 5

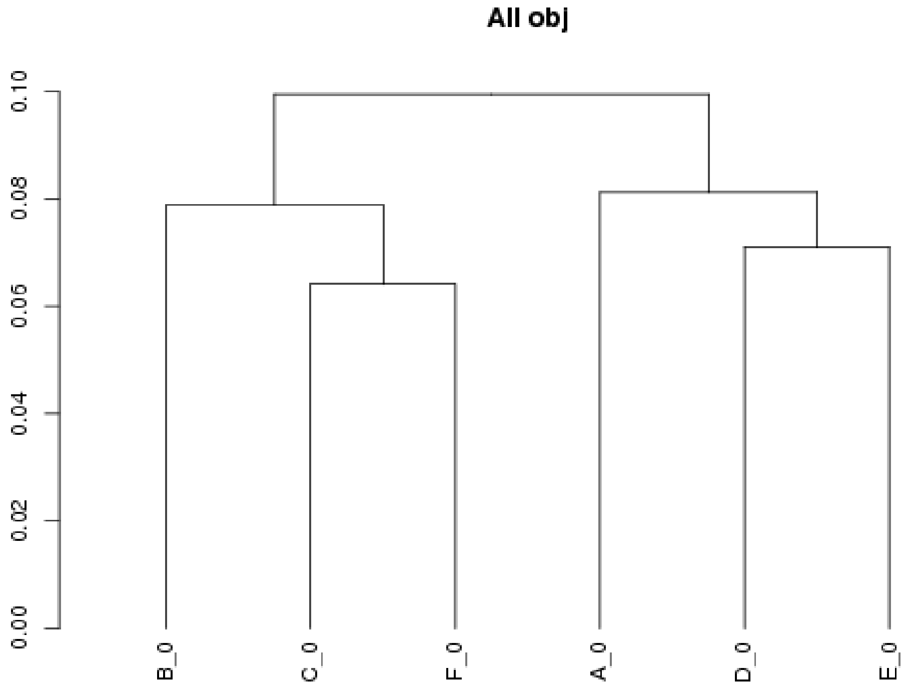

Supplementary Figure 6
